# Transient anabolic effects accompany epidermal growth factor receptor signal activation in articular cartilage *in vivo*

**DOI:** 10.1186/ar4233

**Published:** 2013-05-25

**Authors:** John B Shepard, Jae-Wook Jeong, Nita J Maihle, Sean O'Brien, Caroline N Dealy

**Affiliations:** 1 Center for Regenerative Medicine and Skeletal Development, Department of Reconstructive Sciences, School of Dental Medicine, University of Connecticut Health Center, 263 Farmington Avenue, Farmington CT 06030, USA; 2 Departments of Obstetrics, Gynecology and Reproductive Biology, Michigan State University, 333 Bostwick Avenue NE, Grand Rapids, MI 49503, USA; 3 Departments of Obstetrics and Gynecology, Pathobiology, and Pharmacology, Yale University, 310 Cedar Street, New Haven, CT 06520, USA; 4 Department of Orthopaedic Surgery, School of Medicine, University of Connecticut Health Center, 263 Farmington Avenue, Farmington, CT 06030, USA

**Keywords:** Epidermal growth factor receptor, EGFR, Articular cartilage, Osteoarthritis, Progenitor cells, Chondroprogenitors, Cartilage repair, Mig-6

## Abstract

**Introduction:**

Signals from the epidermal growth factor receptor (EGFR) have typically been considered to provide catabolic activities in articular cartilage, and accordingly have been suggested to have a causal role in osteoarthritis progression. The aim of this study was to determine *in vivo *roles for endogenous EGFR signal activation in articular cartilage.

**Methods:**

Transgenic mice with conditional, limb-targeted deletion of the endogenous intracellular EGFR inhibitor *Mig-6 *were generated using CreLoxP (Mig-6-flox; Prx1Cre) recombination. Histology, histochemical staining and immunohistochemistry were used to confirm activation of EGFR signaling in the articular cartilage and joints, and to analyze phenotypic consequences of *Mig-6 *loss on articular cartilage morphology, proliferation, expression of progenitor cell markers, presence of chondrocyte hypertrophy and degradation of articular cartilage matrix.

**Results:**

The articular cartilage of *Mig-6*-conditional knockout (*Mig-6*-cko) mice was dramatically and significantly thicker than normal articular cartilage at 6 and 12 weeks of age. *Mig-6*-cko articular cartilage contained a population of chondrocytes in which EGFR signaling was activated, and which were three to four times more proliferative than normal *Mig-6*-flox articular chondrocytes. These cells expressed high levels of the master chondrogenic regulatory factor Sox9, as well as high levels of putative progenitor cell markers including superficial zone protein (SZP), growth and differentiation factor-5 (GDF-5) and Notch1. Expression levels were also high for activated β-catenin and the transforming growth factor beta (TGF-β) mediators phospho-Smad2/3 (pSmad2/3). Anabolic effects of EGFR activation in articular cartilage were followed by catabolic events, including matrix degradation, as determined by accumulation of aggrecan cleavage fragments, and onset of hypertrophy as determined by type × collagen expression. By 16 weeks of age, the articular cartilage of *Mig-6*-cko knees was no longer thickened and was degenerating.

**Conclusions:**

These results demonstrate unexpected anabolic effects of EGFR signal activation in articular cartilage, and suggest the hypothesis that these effects may promote the expansion and/or activity of an endogenous EGFR-responsive cell population within the articular cartilage.

## Introduction

Because adult articular cartilage has limited intrinsic regenerative capacity, damage to the tissue due to trauma or long term use during aging is not naturally repaired, causing osteoarthritis [1-3]. Current clinical strategies for articular cartilage repair include cell-based approaches [4], such as Autologous Chondrocyte Implantation [5], in which donor or autologous adult chondrocytes are placed into focal articular cartilage defects; or microfracture [6], in which penetration of the subchondral bone beneath the defect allows influx of endogenous blood and bone marrow cells into the region. A disadvantage of both of these approaches is that the defects tend to be filled by fibrocartilage [7], which lacks the durability of hyaline cartilage. This is likely due to characteristics inherent in the repair cells, which include the poor proliferative capacity of adult or aged chondrocytes, and their tendency to de-differentiate [8]; and the cellular heterogeneity of bone marrow, which contains only a small percentage of progenitor cells capable of chondrogenic differentiation [9,10]. Accordingly, critical steps towards articular cartilage repair and osteoarthritis treatment will be to identify progenitor cells with the ability to form articular cartilage, and to understand the signals that control their proliferation and chondrogenic differentiation [11].

The superficial and/or middle zones of normal articular cartilage have been identified as regions enriched in cells which are highly proliferative and/or which express mesenchymal or progenitor cell markers [12-17]. *In vitro *differentiation assays have demonstrated the potential of these cells to differentiate into the chondrogenic lineage [12-18], and particularly, the permanent hyaline or articular cartilage lineage [12,17,18]. Thus, these populations have been suggested to represent a reserve capacity of the normal articular cartilage for homeostasis or regeneration [14-16].

It is apparent that endogenous progenitors present within the articular cartilage are inadequate for self-repair, as they are observed in osteoarthritic cartilage [14,15,17,19,20]. It has been suggested that advanced age, which is typical of idiopathic osteoarthritis, may reduce the size and/or alter the activity of the progenitor cell pools [19,21,22]. Osteoarthritic cartilage exhibits quantitative and qualitative differences in the expression of progenitor markers compared to normal cartilage [19,20], and cells expressing progenitor markers are markedly more abundant in fetal and juvenile articular cartilage than in articular cartilage from adult or elderly patients [22,23]. Thus, while progenitor cells offer exciting potential for articular cartilage repair and osteoarthritis treatment, there is a critical need to identify signals which promote expansion and/or activity of endogenous progenitor cell pools in the articular cartilage, and/or which stimulate chondrogenic potential by putative exogenous cartilage repair cells.

The epidermal growth factor receptor (EGFR) network is emerging as an important signaling family in cartilage development, homeostasis and disease [24-35]. EGFR signals typically suppress chondrogenic differentiation and/or homeostasis. For example, *in vitro *studies show that EGFR signals suppress initial chondrogenic differentiation by limb mesenchymal cells [27,28], and also suppress matrix synthesis and/or stimulate activity of matrix degradative enzymes by articular chondrocytes [29-32]. EGFR signals also promote the de-differentiation of articular chondrocytes *in vitro *towards fibrogenic cell types [33-35]. Together these observations demonstrate effects of EGFR signaling in suppression of articular cartilage homeostasis, and suggest that activation of EGFR signaling may be a causal factor in osteoarthritis. Consistent with this, EGFR signaling is increased in the articular cartilage of osteoarthritic patients [32], and in rats following experimental surgical osteoarthritis induction [36].

To better understand the function of EGFR signaling in articular cartilage *in vivo*, in this study we have developed a murine model in which activation of EGFR signaling is targeted to the developing and adult limbs, including the joints and articular cartilage, via limb mesoderm-targeted conditional loss of *Mig-6*, an endogenous intracellular inhibitor of EGFR signaling [37]. The articular cartilage of the knee joints of *Mig-6*-cko mice undergoes progressive osteoarthritis-like changes characterized by late-stage articular cartilage degradation, which is unexpectedly preceded by dramatic thickening of the articular cartilage. The articular cartilage of *Mig-6*-cko joints is thickest at six weeks of age, and articular cartilage thickening is preceded by pronounced EGFR signal activation, significantly enhanced proliferation, and expanded expression of the master chondrogenic regulatory factor Sox9 and other markers of putative progenitor cells, which is observed within presumptive articular cartilage as early as postnatal Day 5. Our study demonstrates for the first time anabolic effects in articular cartilage occurring in association with EGFR signal activation, and suggests novel possibilities for future application for cartilage repair and osteoarthritis treatment.

## Materials and methods

### Experimental animals

To produce *Mig-6 *conditional loss targeted to the mesoderm of developing limb buds, the Prx1-Cre transgene, which drives recombination in early limb bud mesenchyme [38], was introduced into *Mig-6*-flox/flox mice [39]. Resultant Prx1-Cre;*Mig-6*-flox/+ male mice were mated with *Mig-6*-flox/flox female mice to obtain *Mig-6 *conditional knockout mice (Prx1-Cre; *Mig-6*-flox/flox). *Mig-6*-flox/flox littermates were used as controls. Genotyping of the mice and embryos was by polymerase chain reaction (PCR) using DNA prepared from tail biopsies. All protocols for animal use were approved by the Animal Care Committee of the University of Connecticut Health Center, and were in accordance with NIH guidelines.

### Histology and staining

Limbs were dissected from adult mice and immediately fixed in 4% paraformaldehyde and processed for paraffin embedding. Histological analysis was performed on 7-μm sections. Safranin O staining of glycosaminoglycans was performed by staining sections with Weigert's Iron Hematoxylin and 0.02% aqueous Fast Green, followed by rinsing with 1% acetic acid and staining with 0.1% aqueous Safranin O.

### Immunohistochemistry

Immunohistochemical staining was performed as previously described [40]. In brief, sections were de-paraffinized, rehydrated and incubated with 3% hydrogen peroxide in water for 15 minutes to quench endogenous peroxidases. After blocking with 10% normal goat serum for rabbit antibodies or M.O.M blocking serum (Vector Laboratories, Burlingame, CA, USA) for mouse antibodies, the slides were incubated with primary antibodies in blocking buffer at 4°C overnight. Dilutions of primary antibodies were as follows: rabbit anti-*Mig-6*, (Sigma, St. Louis, MO, USA), 1:200; rabbit anti-pEGFR (Y1092), (Abcam, Cambridge, MA, USA), 1:250; rabbit anti-SZP (Novus Biologicals, Littleton, CO, USA), 1:100; rabbit anti-Ki67 (Abcam, Cambridge, MA, USA), 1:50; rabbit anti-Notch1 (Abcam), rabbit 1:100; rabbit anti-pSmad2/3 (R&D Systems, Minneapolis, MN, USA), 1:100; anti-Sox9 (Abcam), 1:500; rabbit anti-Aggrecan Neoepitope (Pierce, Rockford, IL, USA), 1:100; mouse anti-collagen type × (Developmental Studies Hybridoma Bank, Iowa City, IA, USA), 1:100; mouse anti-Activated-β-Catenin (Millipore, Temecula, CA, USA), 1:100; goat anti-GDF-5 (R&D Systems), 1:50. The slides were washed with TBS containing 0.1% Tween 20 and then incubated with 1:200 biotinylated goat anti-rabbit IgG (Vector Laboratories) or M.O.M. Biotinylated Anti-mouse Ig Reagent (Vector Laboratories). After washing, the slides were incubated with Vectastain Elite ABC Reagent (Vector Laboratories) and developed with DAB reagent (Vector Laboratories) followed by counterstaining with hematoxylin. For negative controls, the specific antibody was omitted; none showed a positive reaction.

### *In situ *hybridization

The mouse Col10a1 probe (gift of B. Olsen, Harvard University, Boston, MA) was subjected to digoxigenin-labeling (Roche Molecular Biochemicals, Indianapolis, IN, USA) using the protocol described by the manufacturer. *In situ *hybridization was performed on serially sectioned tissue that had been fixed in 4% paraformaldehyde as previously described [40].

### Cell proliferation

Proliferating cells were detected with rabbit anti-Ki67 (Abcam), 1:100. Cell proliferation was quantified using image analysis within Photoshop CS4 Extended (Adobe Systems Inc. San Jose, CA, USA **{AU Query: Please provide the manufacturer's complete name, city, state and country.}**) in fixed areas of 20× digital photographs of adult *Mig-6*-flox/flox and *Mig-6*-cko mice articular cartilage. Ki67-labeled cells within the fixed area were automatically selected by color range, and the number of labeled cells was determined manually using the image analysis 'Count Tool' within Photoshop. Four to six sections of tibial articular cartilage were examined from the knees of five different *Mig-6*-flox/flox and *Mig-6*-cko mice for each time point.

### Determination of articular cartilage thickness

Articular cartilage thickness was determined by measuring the mean distance at its thickest point from the articular cartilage surface to the subchondral bone in Safranin-O stained sections. Images were taken at 40× from representative non-overlapping fields of knees from different *Mig-6*-flox/flox and *Mig-6*-cko mice (*n *= 5 each at 12 weeks; *n *= 6 each at 6 weeks).

### Statistical analysis

Statistical analysis was performed using GraphPad Prism (GraphPad Software Inc., San Diego, USA, USA). For direct comparisons Mann-Whitney U tests were used.

## Results

### Thickening of the articular cartilage of Mig-6-flox;Prx1Cre knee joints

**Figure 1:**
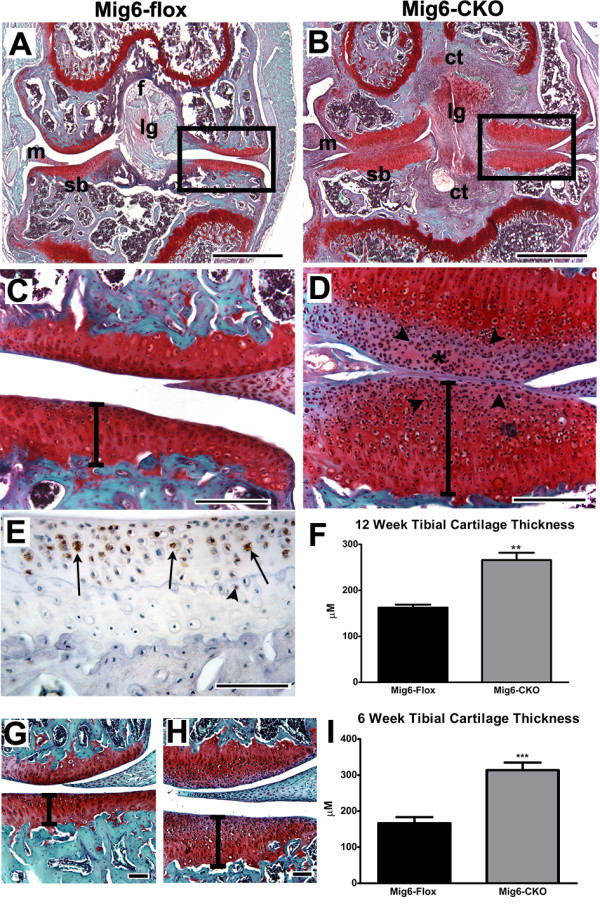
**Articular cartilage thickness and Mig6 localization**. **(A-D) **Sections of 12-week-old normal *Mig-6*-flox (A, C) and *Mig-6-flox;Prx1Cre *conditional knockout (*Mig-6*-cko) knees (B, D) stained with Safranin-O to detect proteoglycan (red) and counterstained with Fast Green (tibia is at the bottom, femur is at the top). The articular cartilage of the tibial and femoral surfaces (boxed areas in A, B, shown at high magnification in C, D) is dramatically thickened in the *Mig-6*-cko knee (B, D). In addition, reduced Safranin-O staining is observed in the the superficial zone of the *Mig-6*-cko articular cartilage (* in D), which is highly cellular and contains numerous rounded chondrocytes often appearing as doublets (arrowheads in D). Note also the thickened ligaments (lg) and menisci (m); abundant connective tissue (ct), and thin subchondral bone (sb) in *Mig-6*-cko knee joint (compare A to B). **(E) **Immunohistochemical detection of *Mig-6 *protein in 12-week-old normal *Mig-6*-flox tibial articular cartilage, showing *Mig-6*-positive chondrocytes (brown stain, arrows) mainly in the superficial zone. Some *Mig-6*-positive chondrocytes were also present in deeper zones (arrowhead) in the articular cartilage adjacent to the tidemark. **(F) **Measurement of the widths of the normal and *Mig-6*-cko tibial articular cartilages (for example, see bars in C, D) shows that the *Mig-6*-cko articular cartilage is dramatically thicker than normal articular cartilage. The articular cartilage of *Mig-6*-cko mice was more than 1.5-fold thicker than normal articular cartilage at 12 weeks of age (*P *<0.01). **(G, H) **Sections of six-week normal *Mig-6*-flox (G) and *Mig-6*-cko knees (H) stained with Safranin-O/Fast Green. The articular cartilage of *Mig-6*-cko joints is also dramatically thickened at six weeks of age (compare bars in G vs. H). **(I) **Measurement of the widths of the normal and *Mig-6*-cko tibial articular cartilages shows that the *Mig-6*-cko articular cartilage is nearly two-fold thicker at six weeks of age (*P *<.001). Scale Bar = 500 μm (A, B); 200 μm (C, D); 100 μm (E, G, H).

Histological analysis of the knee joints of *Mig-6-flox;Prx1Cre *(*Mig-6*-cko) mice revealed dramatic thickening of the articular cartilage (Figure 1). At 12 weeks, the articular cartilage of the tibial surfaces of control *Mig-6*-flox mice was on average 162 ± 15 μm thick (*n *= 5), compared to the average thickness of the tibial articular cartilage of *Mig-6-flox;Prx1Cre *mice, which was 266 ± 36 μm thick (*n *= 5, *P *<0.01, compare Figure 1C vs D and see graph in Figure 1F). The articular cartilage of the femoral surfaces of *Mig-6*-cko joints was also increased (283 ± 19 μm thick for *Mig-6*-cko, *n *= 5, compared to 132 ± 16 μm thick for control *Mig-6*-flox, *n *= 5, *P *<0.01, data not shown). Histochemical staining revealed that Safranin-O positive staining was reduced in the superficial zone of the thickened *Mig-6*-cko articular cartilage (compare Figure 1C, D). The superficial zone of the articular cartilage of the *Mig-6*-cko joints was highly cellular and contained numerous rounded chondrocytes often appearing as doublets (Figure 1D). As shown in Figure 1G and 1H, the articular cartilage of *Mig-6*-cko mice at 6 weeks was also dramatically thickened, and even thicker than at 12 weeks (318 μM + 16.6 μm thick for *Mig-6*-cko, *n *= 6, compared to 170 + 20.9 μm thick for control *Mig-6*-flox, *n *= 6, *P *<0.001, see graph in Figure 1I).

To confirm endogenous expression of *Mig-6 *protein in normal articular cartilage, immunohistochemical staining with a *Mig-6 *antibody was performed, which demonstrated *Mig-6 *protein localization particularly in the superficial zone of the normal 12 week tibial (Figure 1E) and femoral (not shown) knee articular cartilages. Isolated *Mig-6*-positive chondrocytes were also located deep in the articular cartilage adjacent to the tidemark (Figure 1E) and in the subchondral bone (see Additional Figure 1A).

*Mig-6*-cko knee joints also contained thickened lateral and central ligaments which stained intensely with Safranin-O, abundant connective tissue, and enlarged menisci (Figure 1B). The subchondral bone present in the *Mig-6*-cko knee was thin and contained large bone marrow sinuses (Figure 1B).

### EGFR signaling in normal and Mig-6-flox;PrxCre articular cartilage

**Figure 2:**
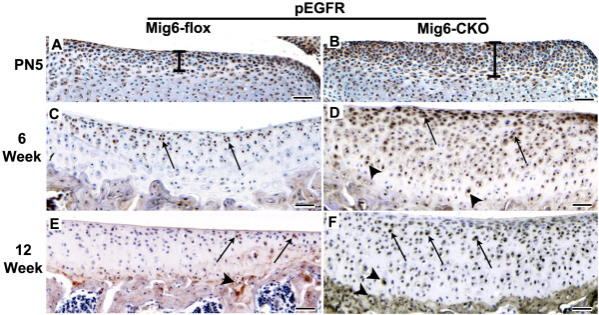
**EGFR signaling**. Sections of the distal tibia of normal *Mig-6*-flox (**A, C, E**) and *Mig-6-flox;Prx1Cre *conditional knockout (*Mig-6*-cko) knees (**B, D, F**) immunostained with a phospho-EGFR antibody (p-EGFR, brown) and counterstained with hematoxylin. At postnatal Day 5 (A, B), p-EGFR signaling is occurring in the distal region of the tibial epiphysis (the presumptive articular cartilage) in both normal and *Mig-6*-cko knees, but staining is more robust in the *Mig-6*-cko knee and the domain of EGFR signal activation is expanded (compare bars in A, B). At six weeks of age (C, D), p-EGFR immunostaining in normal articular cartilage is limited to cells in the superficial zone (arrows in C), but in *Mig-6*-cko articular cartilage staining is intense and abundantly localized in both superficial and middle regions (arrows and arrowheads, respectively, in D). At 12 weeks of age (E, F), p-EGFR-positive chondrocytes are only occasionally observed in the superficial and calcified zones of normal articular cartilage (arrows and arrowheads in E); whereas p-EGFR-positive chondrocytes are present in superficial, middle and calcified zones of the *Mig-6*-cko articular cartilage (arrows and arrowheads in F). Scale Bar = 50 μm (A-F).

Immunostaining with an antibody against the phosphorylated tyrosine residue 1092 of the EGFR kinase domain showed that EGFR signaling was occurring in normal articular cartilage, and increased in *Mig-6*-cko articular cartilage (Figure 2). In normal control *Mig-6*-flox knees, EGFR signaling was activated as early as postnatal Day 5 (the earliest day examined) in chondrocytes located in the distal region of the tibial epiphysis which will form the articular cartilage (Figure 2A). At six weeks of age EGFR signaling in normal tibial articular cartilage was limited to the superficial zone (Figure 2C). In the normal knee at 12 weeks of age, few superficial chondrocytes were EGFR-positive, but EGFR-positive chondrocytes were relatively abundant in the calcified zone adjacent to the chondro-osseous junction, as well as in the subchondral bone itself (Figure 2E). In *Mig-6*-cko knee articular cartilage, EGFR signaling was dramatically enhanced in these regions compared to controls (Figure 2B, D, F). In addition, the domain of EGFR signal activation was expanded as early as postnatal Day 5 (compare bars in Figure 2A vs. Figure 2B), and EGFR-positive chondrocytes were abundant in the middle region of the *Mig-6*-cko articular cartilage at 6 and 12 weeks, a region which in controls contained few EGFR-positive chondrocytes (compare Figure 2C, E to D, F). The patterns of EGFR activation were similar in femoral articular cartilage (not shown).

### Articular chondrocyte proliferation in normal and Mig-6-flox;Prx1Cre knee joints

**Figure 3:**
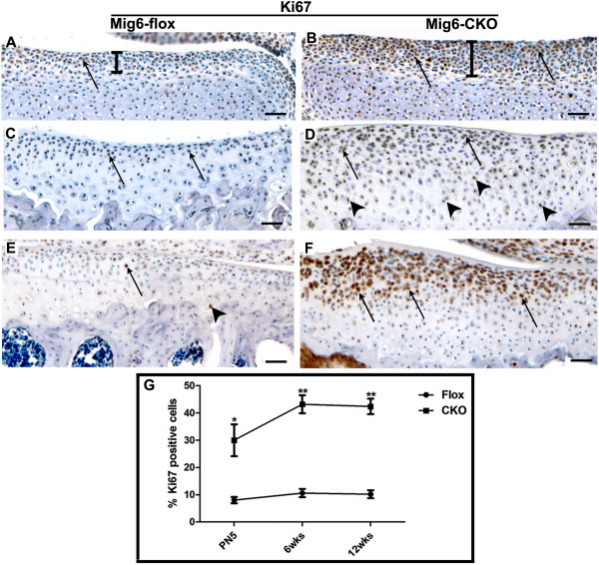
**Cell proliferation**. **(A-F) **Sections of the tibial articular cartilage of normal *Mig-6*-flox (A, C, E) and *Mig-6-flox;Prx1Cre *conditional knockout (*Mig-6-*cko) knees (B, D, F) immunostained with a Ki67 antibody (brown) and counterstained with hematoxylin. (A, C, E) Scattered proliferating cells (arrows in A) are present in the presumptive articular cartilage of normal *Mig-6*-flox tibia at postnatal Day 5 (indicated by bars) and in the superficial zones of normal *Mig-6*-flox tibial articular cartilage at 6 and 12 weeks (arrows in C, E), and rarely in the calcified zone (arrowhead, E). However, in *Mig-6*-cko knees, abundant proliferating cells are located in these regions (arrows in B, D, F), and the domain of robust proliferation is expanded as early as postnatal Day 5 (compare bars in A, B). In addition, proliferating cells are also detected in the middle zone at six weeks (arrowheads in D). **(G) **Cell counting shows that proliferating cells present in normal articular cartilage (closed circles) remain at a low and constant level from Day 5 to 12 weeks while proliferating cells present in *Mig-6*-cko articular cartilage (closed squares) are over three-fold more abundant at postnatal Day 5 (*P *<0.01) and four-fold more abundant at 6 and 12 weeks of age (*P *<0.01). Scale Bar = 50 μm (A-F).

Cell proliferation, as determined by immunostaining with a Ki67 antibody, was dramatically enhanced in the articular cartilage of *Mig-6*-cko knee joints compared to control *Mig-6*-flox knee joints (Figure 3). In control *Mig-6*-flox tibia, only scattered proliferating cells were present in the presumptive articular cartilage at postnatal Day 5 (Figure 3A), and in the articular cartilage at 6 and 12 weeks of age (Figure 3C, E), and quantification of Ki67-positive cells revealed that the level of proliferation remained constant over time (Figure 3G). In contrast, in the *Mig-6*-cko knee, abundant proliferating cells were present in the presumptive articular cartilage at postnatal Day 5, and in the superficial zones at 6 and 12 weeks, and the domain of robust proliferation is expanded as early as postnatal Day 5 (compare bars in Figure 3A vs. Figure 3B). In addition, proliferating cells were also present in deeper regions (Figure 3B, D, F). Cell counting revealed that the number of proliferating cells was about three times higher than controls at postnatal Day 5 (*P *<0.01, *n *= 5, Figure 3G), and four times higher than controls at 6 and 12 weeks of age (*P *<0.01, *n *= 5, Figure 3G).

EGFR signal activation, increased proliferation, and tissue thickening were also observed in other regions of the *Mig-6*-deficient knee joint at six weeks of age (see Additional Figures 1 and 2). These regions include the central ligaments and especially the ligament/cartilage junctions (Additional file 1, Figure S1), as well as the menisci and synovium (see Additional file 2, Figure S2). Endogenous *Mig-6 *immunostaining was present in these tissues in normal six-week *Mig-6*-flox joints, but was not detected in any tissues including the articular cartilage, menisci, bone or ligament of six-week old *Mig-6*-cko joints (see Additional Figures 1 and 2). **{AU Query: The guidelines ask that Additional files/figures be used instead of Supplemental/Supplementary.}**

### Expanded expression of progenitor cell markers in Mig-6-flox;Prx1Cre articular cartilage

**Figure 4:**
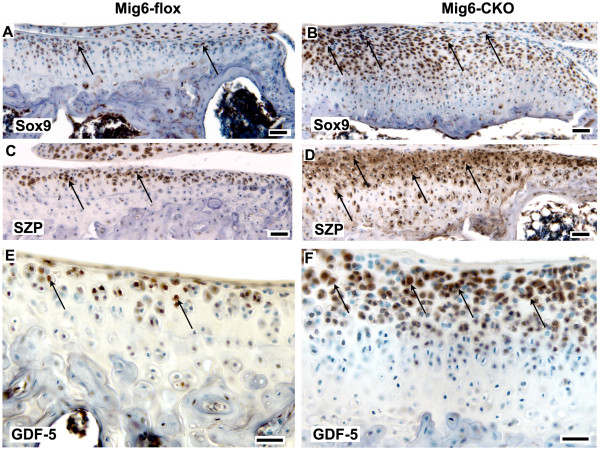
**Localization of Sox9, SZP and GDF-5**. Sections of the tibial articular cartilage of 12-week-old normal *Mig-6*-flox (**A, C, E**) and *Mig-6-flox;Prx1Cre *conditional knockout (*Mig-6*-cko) knees (**B, D, F**) immunostained with antibodies against Sox9 (A, B); superficial zone protein (SZP, C, D); and growth and differentiation factor-5 (GDF5, E, F). In normal articular cartilage, immunostaining is localized to cells mainly in the superficial zone (arrows in A, C, E) while in *Mig-6*-cko articular cartilage, immunostaining is dramatically increased and positive cells are located not only in the superficial zone but also in deeper zones (arrows in B, D, F). Scale Bar = 50 μm (A-D); 25 μM (E, F).

**Figure 5:**
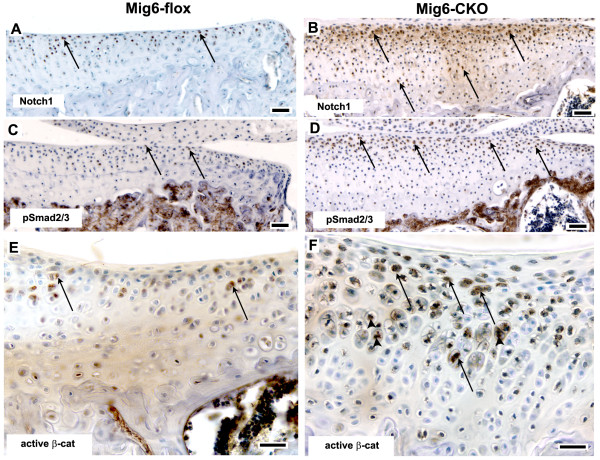
**Localization of Notch1, pSmad2/3 and active β-catenin**. Sections of the tibial articular cartilage of 12-week normal *Mig-6*-flox (**A, C, E**) and *Mig-6-flox;Prx1Cre *conditional knockout (*Mig-6*-cko) knees (**B, D, F**) immunostained with antibodies against Notch1 (A, B); the TGFβ1-mediators phosphorylated Smad2/3 (pSmad2/3) (C, D); and the canonical Wnt mediator activated β-catenin (E, F). In normal articular cartilage, immunostaining is localized to occasional cells mainly limited to the superficial zone (arrows in A, C, E) while in *Mig-6*-cko articular cartilage, immunostaining is intensified and positively-stained cells are located not only in the superficial zone but also in deeper zones (arrows in B, D, F). Strong nuclear localization of active B-catenin is also observed in Mig-6-cko articular chondrocytes (arrowheads in F). Scale Bar = 50 μm (A-D); 25 μM (E, F).

**Figure 6:**
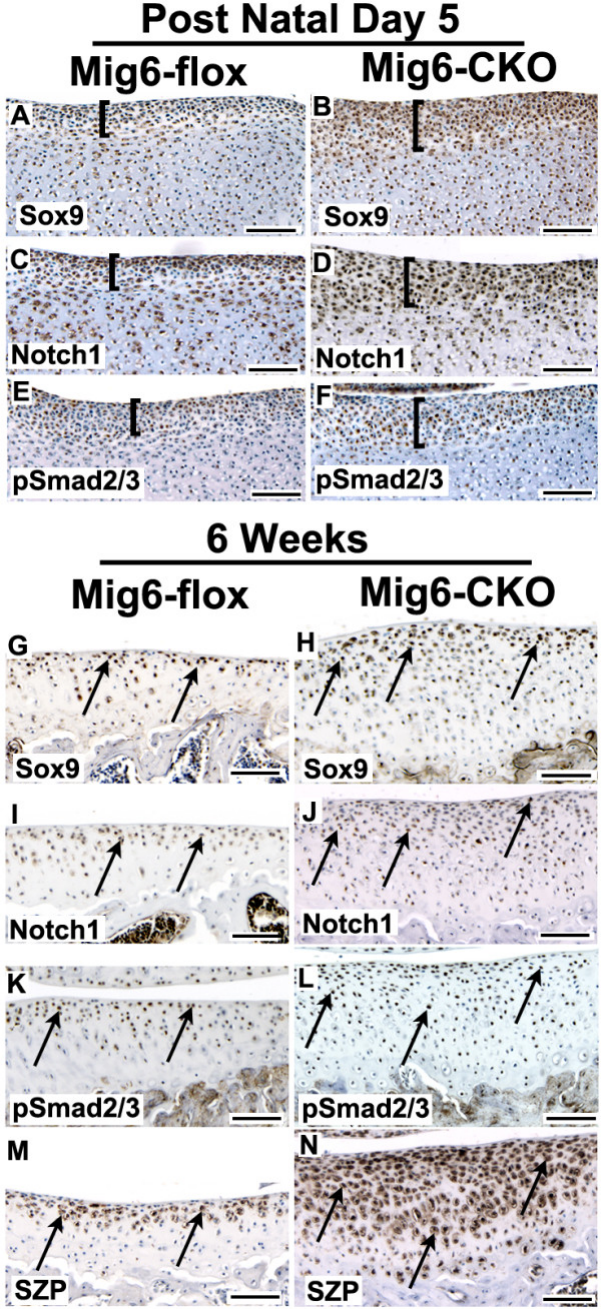
**Time-dependent expansion of marker gene expression**. **(A-F) **Sections of the distal tibial epiphysis of normal *Mig-6*-flox (A, C, E) and *Mig-6-flox;Prx1Cre *conditional knockout (*Mig-6*-cko) (B, D, F) at postnatal Day 5, immunostained with antibodies against Sox9 (A, B); Notch1 (C, D); or the TGFβ1-mediators phosphorylated Smad2/3 (pSmad2/3) (E, F). Immunostaining in the presumptive articular cartilage of the normal *Mig-6*-flox tibia is limited to a distal portion of the epiphyses (indicated by bars in A, C, E). However, immunostaining is intensified in the presumptive articular cartilage of the *Mig-6*-cko tibia, and the region of immunostaining is expanded (compare bars in B, D, F to A, C, E). **(G-N) **Sections of six-week old tibial articular cartilage of normal *Mig-6*-flox (**G, I, K, M**) and *Mig-6*-cko (**H, J, L, N**) joints immunostained with antibodies against Sox9 (G, H); Notch1 (I, J); pSmad2/3 (K, L) or superficial zone protein (SZP, M, N). Enhanced marker expression by cells in the superficial and middle zones is evident in the six-week-old *Mig-6*-cko articular cartilage compared to control *Mig-6*-flox articular cartilage (arrows). Scale bar = 100 nm.

As shown by immunostaining, the relative abundance of cells expressing Sox9, superficial zone protein (SZP), growth and differentiation factor-5 (GDF-5), Notch1, activated β-catenin, and the transforming growth factor beta (TGF-β) mediators phospho-Smad2/3 (pSmad2/3), was markedly increased in *Mig-6*-cko articular cartilage compared to control articular cartilage (Figures 4 and 5). At 12 weeks of age, cells expressing these markers were present in the superficial zone of control *Mig-6*-flox tibial articular cartilage (Figure 4A, C, E and Figure 5A, C, E). However, in 12-week old *Mig-6*-cko tibial articular cartilage, cells expressing these markers were considerably more abundant and were present not only in the superficial but also in the middle zones (Figure 4B, D, F and Figure 5B, D, F). The distribution and relative abundance of these markers in *Mig-6*-cko femoral cartilage was also increased compared to control *Mig-6*-flox femoral articular cartilage (not shown). At six weeks of age, enhanced expression and expanded distribution of Sox9, Notch1, pSmad2/3 and SZP was also evident in *Mig-6*-cko articular cartilage (Figure 6H, J, L, N) compared to control *Mig-6*-flox articular cartilage (Figure 6G, I, K, M). Notably, an increased abundance and expanded distribution of cells expressing of Sox9, Notch1 and pSmad2/3 protein relative to controls was also detected in the presumptive articular cartilage of *Mig-6*-cko at postnatal Day 5, the earliest day examined (compare region shown by bars in *Mig-6*-flox (Figure 6A, C, E) vs *Mig-6*-cko (Figure 6B, D, F). Measurement of the length of the bars indicates the region of expanded marker gene expression in the *Mig-6*-cko is approximately 25% thicker than in normal *Mig-6*-flox controls.

### Matrix remodeling and chondrocyte hypertrophy in Mig-6-flox;Prx1Cre articular cartilage

**Figure 7:**
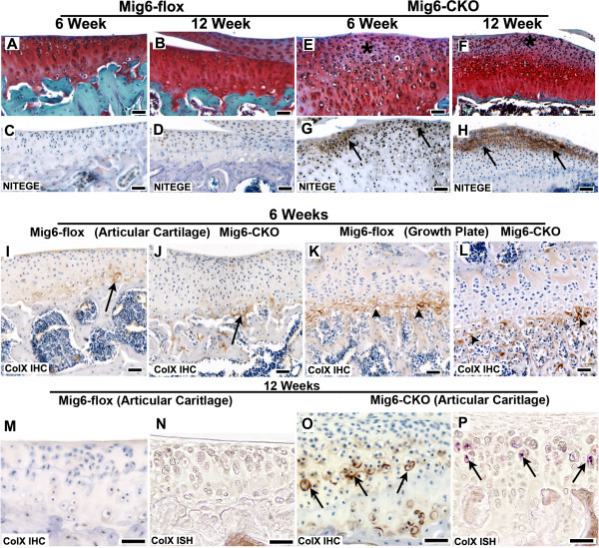
**Matrix remodeling and chondrocyte hypertrophy**. **(A-D) **Sections of the tibial articular cartilage of 6-week-old (A, C) and 12-week-old (B, D) normal *Mig-6*-flox knees stained with Safranin-O (A, B) or immunostained for NITEGE-positive aggrecan-cleavage fragments (NITEGE, C, D). The normal articular cartilage stains uniformly for Safranin-O, and aggrecan cleavage fragments are not detected. **(E-H) **Sections of the tibial articular cartilage of 6-week-old (E, G) and 12-week-old (F, H) *Mig-6-flox;Prx1Cre *(*Mig-6*-cko) knees stained with Safranin-O (E, F) or immunostained for NITEGE-positive aggrecan-cleavage fragments (NITEGE, G, H). The superficial zone of *Mig-6*-cko knee articular cartilage stains weakly for Safranin-O (* in E, F) and contains NITEGE-positive aggrecan cleavage fragments (arrows in G, H) which are more abundant at 12 weeks than at 6 weeks (compare H to G). **(I-L) **Sections of the tibial articular cartilage (I, J) of six-week-old normal *Mig-6*-flox (I) and *Mig-6-flox*-cko (J) knees, immunostained with a collagen type × antibody to identify hypertrophic chondrocytes. Only an occasional hypertrophic chondrocyte is detected in either normal or *Mig-6*-cko articular cartilage at six weeks of age (arrows in I, J). As a positive control (K, L) note the robust collagen type × immunostaining in the hypertrophic chondrocytes of both normal *Mig-6*-flox (K) and *Mig-6*-cko growth plates (arrowheads in K, L). **(M-P) **Sections of the tibial articular cartilage of 12-week-old normal *Mig-6*-flox (M, N) and *Mig-6-flox*-cko (O, P) knees, immunostained with a collegen type × antibody (M, O) or subjected to *in situ *hybridization for type × collagen mRNA (N, P). While few or no hypertrophic chondrocytes are detected in normal articular cartilage, several hypertrophic chondrocytes are observed in *Mig-6*-cko articular cartilage (arrows in O, P). Scale Bar = 50 μm (A-H); 20 μm (I-P).

Little or no matrix turnover, as determined by immunostaining with an antibody to the aggrecan-cleavage fragment NITEGE, was detected in normal *Mig-6*-flox tibial articular cartilage at 6 and 12 weeks of age (Figure 7A-D). Safranin-O staining in normal *Mig-6*-flox tibial articular cartilage was also uniform at 6 and 12 weeks. In contrast, Safranin-O staining was reduced in the superficial zone of *Mig-6*-cko tibial articular cartilage, and this region contained immunoreactive NITEGE cleavage fragments (Figure 7E-H). The intensity of NITEGE immunostaining at 6 weeks was low, and became considerably increased by 12 weeks in *Mig-6*-cko articular cartilage (compare Figure 7G to H). Little or no NITEGE-positive immunostaining was observed in either normal or Mig-6-deficient presumptive articular cartilage at postnatal Day 5 (not shown). Few hypertrophic chondrocytes, detected through immunostaining for type × collagen and/or by *in situ *hybridization with a type × collagen probe, were observed in the articular cartilage of either normal *Mig-6-*flox or *Mig-6*-cko knees at six weeks (Figure 7I-L). However, at 12 weeks, while few hypertrophic chondrocytes were detected in normal *Mig-6-*flox knees, several hypertrophic chondrocytes were observed in the articular cartilage of *Mig-6*-cko knees (compare Figure 7M, N to Figure 7O, P).

### Late stage degradation in Mig-6-flox;Prx1Cre articular cartilage

**Figure 8:**
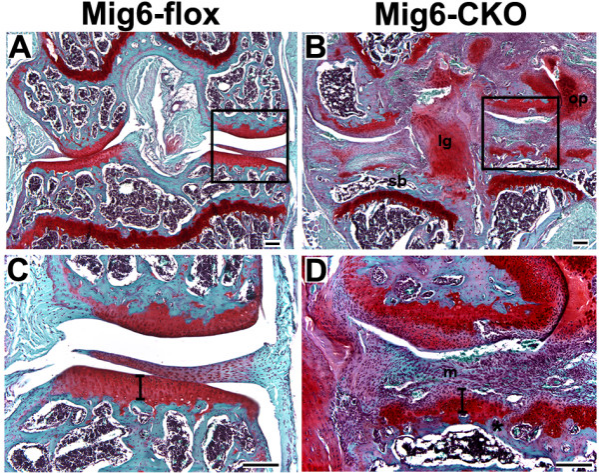
**Late-stage articular cartilage degradation**. Sections of 16-week normal *Mig-6*-flox (**A, C**) and *Mig-6-flox;Prx1Cre *conditional knockout (*Mig-6*-cko) knees (**B, D**) stained with Safranin-O and counterstained with Fast Green (tibia is at the bottom, femur is at the top; boxed areas in A, B are enlarged in C, D). At 16 weeks, the tibial articular cartilage of the *Mig-6*-cko knee is comparable in thickness to that of the normal knee (bars in C, D) but is discontinuous, with loss of surface integrity (* in D) and fusion with the overlying fibrogenic meniscal tissue (m). In addition, *Mig-6*-cko knee joints contain fused central ligaments which stain intensely with Safranin-O (lg in B), prominent osteophytes (op), and thin subchondral bone (sb). Scale Bar = 100 μm.

At 16 weeks of age, *Mig-6*-cko articular cartilage was no longer overtly thickened and degradation of the articular cartilage along with gross joint abnormality was present (Figure 8). The tibial articular cartilage of *Mig-6*-cko knee joints at 16 weeks was comparable in thickness to normal articular cartilage at that age (Figure 8), but was reduced in thickness compared to *Mig-6*-cko articular cartilage at 12 and 6 weeks of age (compare Figure 8D to Figures 1D, H). In addition, the tibial articular cartilage was discontinuous, with loss of integrity both at the surface and at the chondro-osseous junction (Figure 8D). In some regions of the joint, it was not possible to detect a clear separation between the tibial articular cartilage surface and the meniscal fibrous tissue that filled the inter-articular space (Figure 8D). The knee joints of 16-week-old *Mig-6*-cko mice also contained fused and highly chondrified central ligaments; thickened and fibrogenic menisci; reduced subchondral bone area; and prominent central and lateral osteophytes (Figure 8B).

## Discussion

As EGFR signals have typically been reported to have negative roles in cartilage differentiation and homeostasis [27-36], our observation that *in vivo *activation of EGFR signaling leads to transient thickening of the articular cartilage is unexpected, and suggests potential novel anabolic functions for EGFR signals in cartilage tissue. The articular cartilage thickening that accompanies EGFR activation is also accompanied by increased proliferation of cells within the articular cartilage. EGFR signals have well-established mitogenic roles for many progenitor cell types, including mesenchymal progenitors [41,42], and our previous studies have shown that EGFR signals stimulate *in vitro *and *in vivo *proliferation by embryonic limb mesenchymal cells [27,43], and are also required for *in vivo *proliferation of immature chondrocytes in developing limb skeletal elements [44]. As proliferation is a requirement for chondrogenic differentiation by progenitor cells [45], our observation that activation of EGFR signaling stimulates proliferation in the articular cartilage, and especially in the superficial layers, which are enriched in progenitor cells [13-17], is consistent with an important role for endogenous EGFR signals in providing these pro-proliferative cues.

Progenitor cell populations present in the articular cartilage have been identified based on their expression of cell surface mesenchymal progenitor markers [14,15]; and/or expression of Notch1, Sox9, superficial zone protein (SZP) (aka prg4/lubricin), and growth and differentiation factor-5 (GDF-5) [12,13,16-18,46], which have been implicated in cartilage or articular cartilage lineage differentiation, and/or maintenance of chondrogenic potential [16,18,46-48]. Although definitive markers for articular cartilage progenitors are lacking [19], our observation that *Mig-6*-deficient articular cartilage contains a population of cells which are highly proliferative and which express Notch1, Sox9, SZP and GDF-5 suggests the existence of an endogenous EGFR-responsive progenitor cell pool in articular cartilage. These putative progenitor cells appear to have the potential to differentiate and contribute to the thickened *Mig-6*-cko articular cartilage, as the chondroprogenitor marker and master chondrogenic regulator, Sox9, is up-regulated by the cells as they transition from the superficial zone into deeper regions of the articular cartilage.

The EGFR-responsive putative progenitor cells we observe in *Mig-6*-deficient articular cartilage also express increased levels of the TGF-β mediators pSmad2/3, as well as high levels of nuclear-localized activated β-catenin, suggesting TGF-β and canonical Wnt signaling pathways are stimulated in these cells. This is consistent with the proposed roles for these pathways as key regulators of articular cartilage progenitor cell and/or articular chondrocyte phenotypes [18,49]. For example, *in vitro*, articular cartilage superficial zone cells have been shown to proliferate and express progenitor or superficial zone markers in response to TGF-β1 [50] and to transient activation of canonical Wnt signaling [18]; and *in vivo*, transient activation of β-catenin signaling, which like the EGFR has typically been associated with osteoarthritis [51] also causes articular cartilage thickening in postnatal mice [52]. Intriguingly, synergistic interactions occur among the TGF-β, Wnt and EGFR network in other systems [53-57]. The co-localization of pSmad2/3 and activated β-catenin by cells in the Mig-6-cko articular cartilage in which EGFR signaling is also activated suggests that expansion or activation of putative progenitor cells within the articular cartilage may involve interactions between the EGFR network and the TGF-β and canonical Wnt networks.

*Mig-6 *is an intracellular inhibitor of EGFR signaling [37] which binds to the intracellular kinase domain of the EGFR [58]. One of the roles of *Mig-6 *is as a tumor suppressor gene [59], and in accordance with the well-established involvement of EGFR signaling in oncogenic progression [60], mice with global *Mig-6 *loss experience widespread and precocious tumor development [61]. Thus, it has been suggested that *Mig-6 *mediated inhibition of EGFR signals has evolved to control potentially inappropriate proliferative responses following cellular injury or stress [59]. Notably, *Mig-6 *is up-regulated in response to mechanical stress [62], and mice with global *Mig-6 *loss have previously been reported to develop early-onset degenerative joint disease in their load-bearing joints [26]. The reported knee joint phenotype of mice with global *Mig-6 *loss is similar to what we have observed in *Mig-6*-cko mice, including the presence of fibrous tissue and osteophytes within the joint, and loss of proteoglycan staining and eventual degradation of the articular cartilage [26]. The present study extends these findings by revealing previously unsuspected anabolic effects accompanying *Mig-6 *loss and EGFR signal activation in articular cartilage, and by suggesting the presence of a putative progenitor cell population in the articular cartilage that is expanded in response to *Mig-6 *loss. Our observations suggest that release of *Mig-6*-mediated inhibition of EGFR signaling in *Mig-6*-cko articular cartilage activates EGFR-mediated anabolic responses by stimulating the proliferation and expansion of what we suggest are progenitor populations within the articular cartilage.

It is important to point out that as *Mig-6 *functions are downstream of ligand activation of the EGFR, *Mig-6 *loss does not result in constitutive or ligand-independent EGFR activation, but rather represents de-repression of endogenous ligand-bound receptor signals [37,58]. The endogenous expression of *Mig-6 *in chondrocytes, mainly in the superficial zone of normal adult murine articular cartilage, closely matches that of endogenous EGFR signaling, and is consistent with activation of EGFR signaling in this region following *Mig-6 *loss. As few *Mig-6*-positive chondrocytes were detected outside the superficial zone in normal articular cartilage, it is possible that the enhanced EGFR signal activation we observed in deeper regions of the *Mig-6*-cko articular cartilage is due to release of *Mig-6*-inhibition by cells expressing *Mig-6 *near or below the limit of immunohistochemical detection, and/or is the result of proliferative expansion of the superficial zone cells which originally expressed it. The three- to four-fold increase in proliferative rate by superficial and middle zone cells in *Mig-6*-cko articular cartilage is consistent with this latter possibility.

The nature of the endogenous ligand-receptor interactions mediating the EGFR responses we have observed in *Mig6*-deficient articular cartilage is unknown. For example, while the EGFR ligands transforming growth factor alpha (TGF-α), and EGF are expressed by articular chondrocytes [32,63], studies typically implicate their functions in catabolic effects of EGFR signaling associated with osteoarthritic damage [29-32], rather than the anabolic effects we have observed here. As distinct EGFR signal outputs may be generated by differential ligand activation [64], it is possible that anabolic EGFR activities could be mediated by ligands other than EGF or TGF-α; alternately, anabolic vs. catabolic EGFR activities in articular cartilage could be related to differences in the timing or level of EGFR activation achieved in *in vitro *studies vs. our *in vivo *studies. Choice of heterodimerization partner within the EGFR network can also influence signal output [65], indicating additional involvement from other EGFR-related receptors could also occur. In addition, *Mig-6 *can directly bind to and inhibit signal transduction by the EGFR-related receptor, ErbB2 [66]. Some EGFR-independent effects of *Mig-6 *have been reported including direct inhibition of ERK [67] and hepatocyte growth factor (HGF)/Met signaling [68]; however, HGF is not a potent regulator of anabolic or catabolic gene expression in articular chondrocytes [69]. Our observation that EGFR signaling is dramatically increased in *Mig-6*-cko articular cartilage in the same regions where we observe major phenotypic effects is consistent with a potentially primary role for the EGFR in mediating most, if not all, of the articular cartilage responses we have observed.

The catabolic effects of EGFR signaling in mature articular chondrocytes *in vitro *include de-differentiation towards fibrogenic cell types [33-35]. Conceivably then, a possible explanation for the thickening of the *Mig-6*-cko articular cartilage could be that EGFR signal activation results in de-differentiation and proliferation of mature articular chondrocytes. However, we favor a view that articular cartilage thickening in *Mig-6*-cko mice results from stimulation of an endogenous progenitor cell response, rather than a de-differentiative response by mature cells. In support of this view are our observations that enhanced EGFR signal activation, increased proliferation, and expanded expression of progenitor cell markers, occur as early as postnatal Day 5, at which stage the articular cartilage is not morphologically distinct and is considered immature. Indeed, at postnatal Day 5, the presumptive articular cartilage consists only of a superficial layer, and the middle and deeper zones are not yet formed [70]. Thus, we believe it is very likely that the time-dependent thickening of *Mig-6*-cko articular cartilage is due to expansion and proliferation of an endogenous EGFR-responsive progenitor population present in the articular cartilage and especially the superficial zone. If true, this would suggest previously unsuspected activities for EGFR signaling in promoting progenitor cell responses in articular cartilage, which could have important potential utility for cartilage repair and regenerative medicine.

Ultimately, catabolic effects of sustained EGFR activation in *Mig-6*-cko articular cartilage predominate over anabolic ones, eventually causing thinning, loss of integrity and degradation of the articular cartilage. One possible explanation for these degenerative changes is that the immature cartilage matrix present in the articular cartilage surface layer may be insufficient to withstand cumulative loading to the joints. It is also possible that the increased matrix enzyme activity in *Mig-6*-cko articular cartilage we have observed eventually outpaces deposition of new matrix by the EGFR-responsive progenitor-derived cells. Indeed, sustained matrix degradation is considered to be a turning point in osteoarthritic progression leading to irreversible cartilage damage [71]. Consistent with this possibility, high-level activation of matrix enzymes occurs in the *Mig-6*-cko articular cartilage at 12 weeks, shortly before overt degradation and thinning of the articular cartilage. Activation of chondrocyte hypertrophy in the articular cartilage is also considered to be part of the disease pathology leading to articular cartilage degeneration [72]. Consistent with this, hypertrophic chondrocytes are observed in *Mig-6*-cko articular cartilage, but not in normal *Mig-6-*flox articular cartilage, at 12 weeks of age, shortly before overt degradation of the articular cartilage occurs. These observations suggest the hypothesis that EGFR signal activation has dual effects in articular cartilage, including an initial anabolic stimulation mediated by expansion of progenitor cells, which is followed by inappropriate activation of matrix remodeling and chondrocyte hypertrophy, leading to articular cartilage degradation and overt joint disease. It is important to point out that at six weeks of age, which is when the Mig-6-cko articular cartilage is thickest, and proliferation is greatest, hypertrophic chondrocytes are not yet detected. This suggests that anabolic effects of EGFR signal activation precede catabolic ones, and are not necessarily coincident. Accordingly, an intriguing consideration is the possibility that *transient *activation of EGFR signaling might result in stimulation of anabolic activities, perhaps without catabolic ones, which could suggest novel future utility for EGFR signal activation in strategies for articular cartilage repair and osteoarthritis treatment. Additional studies are needed to clarify whether anabolic effects resulting from EGFR activation can result in formation of functional articular cartilage tissue.

## Conclusions

Our study provides *in vivo *evidence for the involvement of EGFR signal activation in regulating potentially distinct anabolic and catabolic activities in articular cartilage, and demonstrates that the intracellular inhibitor *Mig-6 *normally functions to limit these activities. Release of *Mig-6*-mediated inhibition of EGFR signals leads to an initial, transient, thickening of the articular cartilage accompanied by proliferation and expansion of an EGFR-responsive cell population, which expresses high levels of the master chondrogenic regulatory factor Sox9, as well as high levels of other putative progenitor markers. In the presence of sustained EGFR activation, these anabolic effects are followed subsequently by accelerated catabolic effects (matrix degradation and hypertrophy) which may contribute to the eventual loss of the articular cartilage in this model.

## Abbreviations

EGF: epidermal growth factor; EGFR: epidermal growth factor receptor; GDF-5: growth and differentiation-5; HGF: hepatocyte growth factor; HB-EGF: heparin-binding epidermal growth factor; *Mig-6*-cko: *Mig-6 *conditional knock out; pSmad2/3: phospho-Smad2/3; SZP: superficial zone protein; TGF-α: transforming growth factor alpha; TGF-β: transforming growth factor beta

## Competing interests

CD is a principal in a University of Connecticut faculty start-up called Chondrogenics, Inc. The goal of this company is to develop the potential of human embryonic and induced pluripotent stem cells for osteoarthritis treatment. Chondrogenics is funding a subcontract to CD's lab at the University of Connecticut to perform studies related to the company's goal. CD does not receive any direct salary, fee or reimbursement from the company. Chondrogenics has not funded the research described in this manuscript. However, Chondrogenics could benefit in the future from the conceptual information described in this manuscript, and is contributing to the cost for publication of this manuscript. No other authors declare competing interests.

## Authors' contributions

JS participated in acquisition, analysis and interpretation of data, and in manuscript preparation. JJ and NJM participated in data interpretation and manuscript preparation. SO participated in data acquisition and analysis. CND participated in experimental concept and design, data analysis and interpretation, and manuscript preparation. All authors read and approved the final manuscript.

## Supplementary Material

Additional Figure 1 **Mig6 expression, EGFR activation and proliferation in ligament and bone**. Sections of the center of normal six-week old *Mig-6*-flox knees (**A, C, E**) and *Mig-6-flox;Prx1Cre *conditional knockout (*Mig-6*-cko) knees (**B, D, F**) immunostained with antibodies against *Mig-6 *(A, B); phosphorylated EGFR (p-EGFR, C, D); or Ki67 (E, F). (A, B) Endogenous localization of *Mig-6 *protein in ligament (arrows in A) and bone (arrowheads in A) which is absent in the knockout animal (B). (C-F) EGFR signaling and proliferation are occurring within the ligament especially the ligament/cartilage junction (arrows), and within the subchondral bone (arrowheads), of the normal *Mig-6*-flox knee (C, E), and EGFR signaling and proliferation are increased in these tissues in the *Mig-6-*cko knee (compare D, F to C, E). Scale Bar = 100 μm. Click here for file

Additional Figure 2 **Mig6 expression, EGFR activation and proliferation in menisci and/or synovium**. Sections of the menisci of normal six-week-old *Mig-6*-flox knees (**A, C, E**) and *Mig-6-flox;Prx1Cre *conditional knockout (*Mig-6*-cko) knees (**B, D, F**) immunostained with antibodies against *Mig-6 *(A, B); phosphorylated EGFR (p-EGFR, C, D); or Ki67 (E, F). (A, B) Endogenous localization of *Mig-6 *protein in the superficial region of the meniscus (arrowheads in A) which is absent in the knockout animal (B). (C-F) EGFR signaling and proliferation are occurring within the superficial region of the meniscus (arrowheads) of the normal *Mig-6*-flox knee (C, E), and EGFR signaling and proliferation is enhanced and extends further into the meniscus in the *Mig-6-*cko knee (compare D, F to C, E). In addition, immunostaining for pEGFR is also present in the thickened Mig-6-cko synovial tissue (arrow in D). Scale Bar = 100 μm. Click here for file
