# Supplementary figures and images for: Transient anabolic effects accompany epidermal growth factor receptor signal activation in articular cartilage in vivo
Source: Arthritis Res Ther. 2013 May 25;15(3):R60. doi: 10.1186/ar4233 (PMC4060279; doi:10.1186/ar4233)

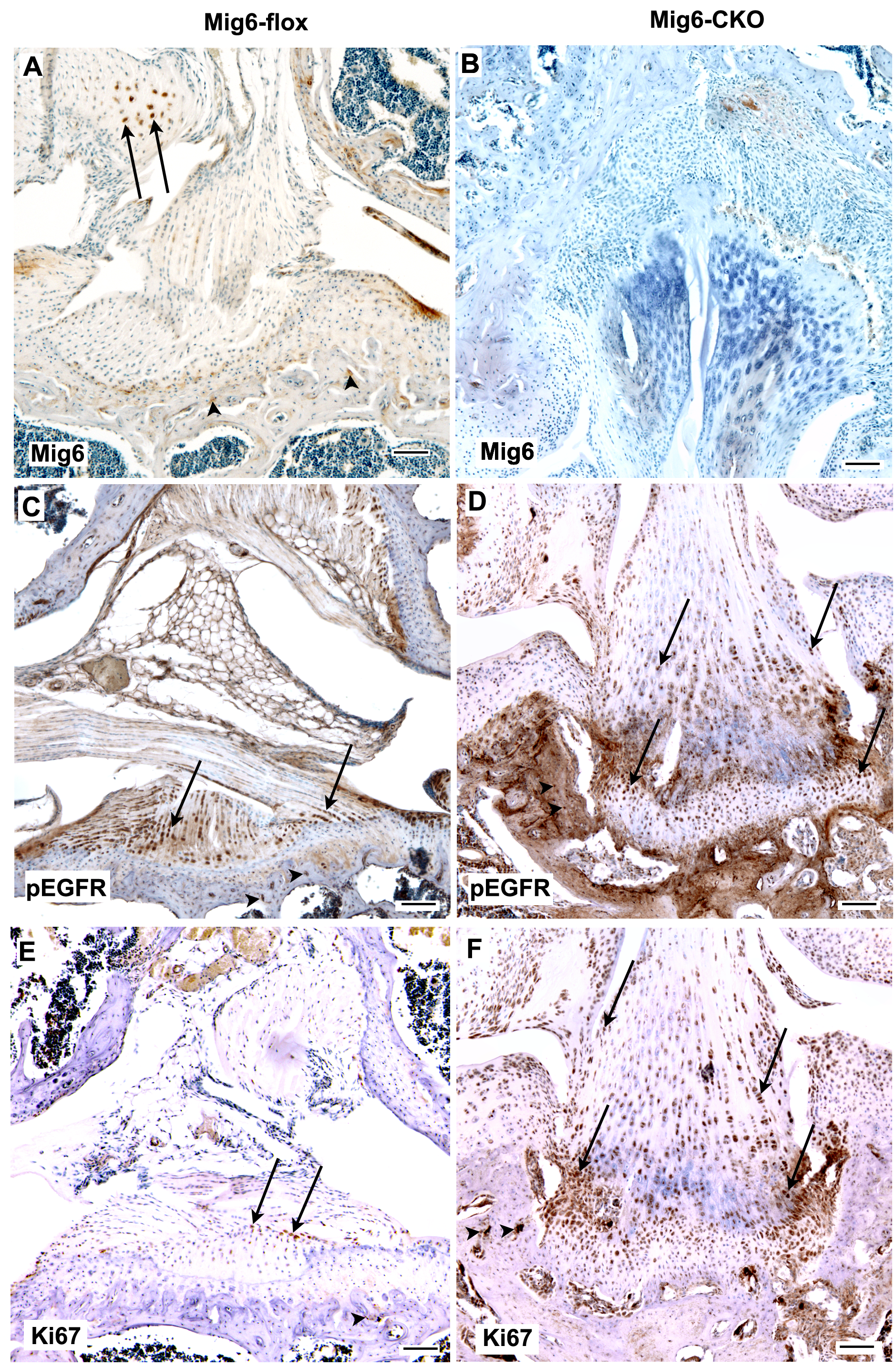

Supplement: Additional Figure 1 — Mig6 expression, EGFR activation and proliferation in ligament and bone. Sections of the center of normal six-week old Mig-6-flox knees (A, C, E) and Mig-6-flox;Prx1Cre conditional knockout (Mig-6-cko) knees (B, D, F) immunostained with antibodies against Mig-6 (A, B); phosphorylated EGFR (p-EGFR, C, D); or Ki67 (E, F). (A, B) Endogenous localization of Mig-6 protein in ligament (arrows in A) and bone (arrowheads in A) which is absent in the knockout animal (B). (C-F) EGFR signaling and proliferation are occurring within the ligament especially the ligament/cartilage junction (arrows), and within the subchondral bone (arrowheads), of the normal Mig-6-flox knee (C, E), and EGFR signaling and proliferation are increased in these tissues in the Mig-6-cko knee (compare D, F to C, E). Scale Bar = 100 μm. [file ar4233-S1.TIFF]

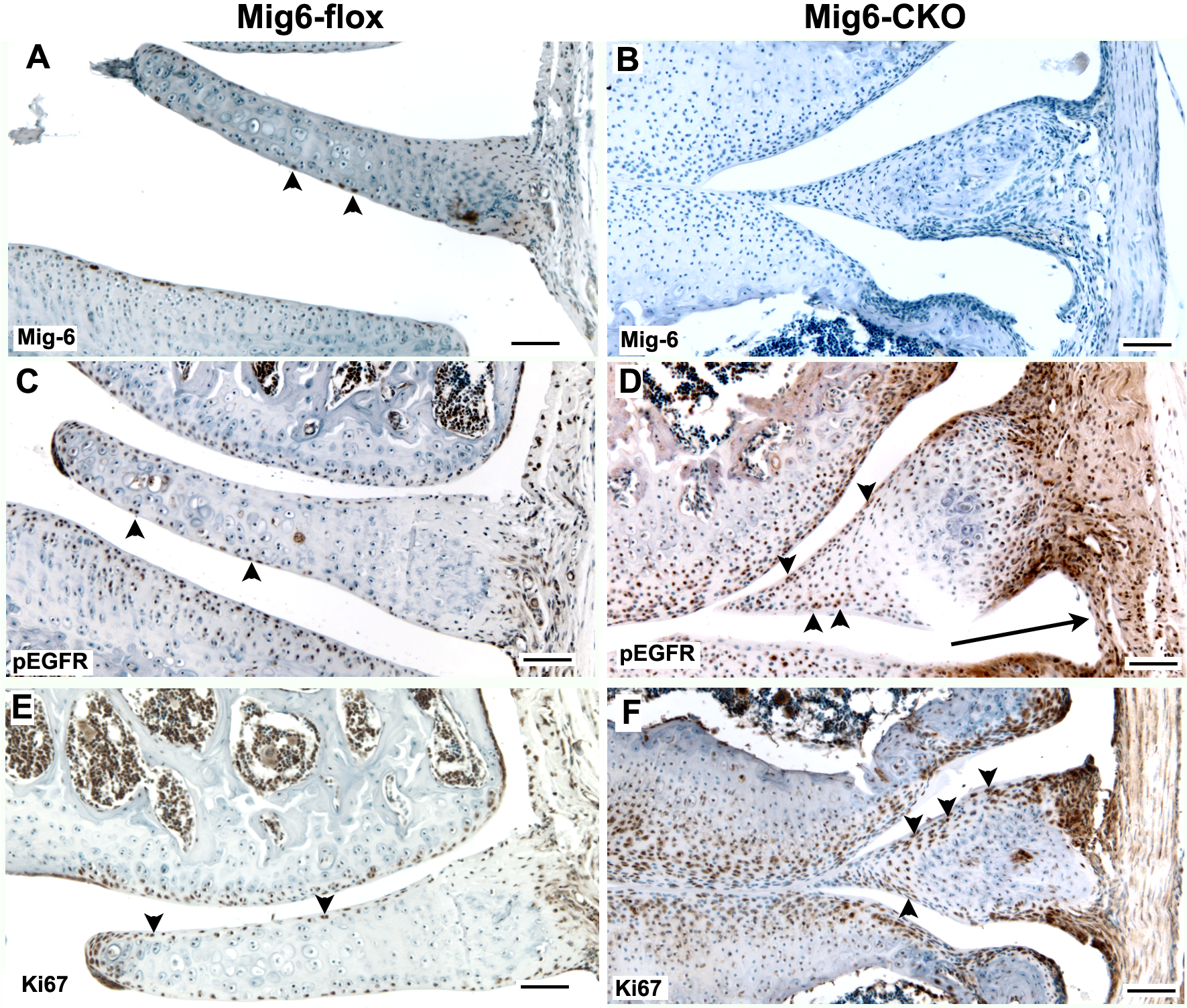

Supplement: Additional Figure 2 — Mig6 expression, EGFR activation and proliferation in menisci and/or synovium. Sections of the menisci of normal six-week-old Mig-6-flox knees (A, C, E) and Mig-6-flox;Prx1Cre conditional knockout (Mig-6-cko) knees (B, D, F) immunostained with antibodies against Mig-6 (A, B); phosphorylated EGFR (p-EGFR, C, D); or Ki67 (E, F). (A, B) Endogenous localization of Mig-6 protein in the superficial region of the meniscus (arrowheads in A) which is absent in the knockout animal (B). (C-F) EGFR signaling and proliferation are occurring within the superficial region of the meniscus (arrowheads) of the normal Mig-6-flox knee (C, E), and EGFR signaling and proliferation is enhanced and extends further into the meniscus in the Mig-6-cko knee (compare D, F to C, E). In addition, immunostaining for pEGFR is also present in the thickened Mig-6-cko synovial tissue (arrow in D). Scale Bar = 100 μm. [file ar4233-S2.TIFF]
